# A new species of *Neoergasilus* Yin 1956 (Copepoda: Cyclopoida: Ergasilidae) parasitic on the catfish *Clarias gariepinus* (Burchell, 1822) (Siluriformes: Clariidae) from South Africa

**DOI:** 10.1007/s11230-024-10189-6

**Published:** 2024-09-24

**Authors:** Precious P. Fikiye, Liesl L. Van As, Marliese Truter, Nico J. Smit, Kerry A. Hadfield

**Affiliations:** 1 Water Research Group, Unit for Environmental Sciences and Management, North-West University, Private Bag X6001, Potchefstroom, 2520 South Africa; 2 Department of Zoology and Entomology, University of the Free State, P.O. Box 339, Bloemfontein, 9300 South Africa; 3 South African Institute for Aquatic Biodiversity, Private Bag 1015, Makhanda, 6140 South Africa

## Abstract

Twenty-one specimens of an ergasilid were collected from the gills of the sharptooth catfish, *Clarias gariepinus* (Burchell), from the Eastern Cape, South Africa. The first leg and antennae morphology conformed to the genus *Neoergasilus* Yin, 1956. While the invasive *Neoergasilus japonicus* (Harada, 1930) is the only *Neoergasilus* species that has been reported from Africa, the combination of several characteristics, separates the Eastern Cape specimens from *N. japonicus* and the other eight *Neoergasilus* congeners. These include an inflated cephalothorax; the presence of an oval dorsal ornamentation anterior to the cephalosome; a spine on the posterodistal margin of the first antennal segment; a cone-like process at the proximal margin of the second antennal segment; a knob-like process on the inner distal margin of the first exopodal segment of leg 1 and two forked spines on the third exopodal segment; leg 4 bearing a 2-segmented exopod and 3-segmented endopod; a single-segmented fifth leg with a seta extending from the base of the pedigerous somite and three unequal setae on its free segment; and a median caudal rami seta with an array of spines. Supporting genetic data were generated using two partial ribosomal RNA genes, 18S and 28S, and one partial mitochondrial DNA gene, COI. The Eastern Cape species is here proposed as new to science and described as *Neoergasilus africanus* **n. sp.** (Ergasilidae: Cyclopoida). This is the first *Neoergasilus* species described from the sharptooth catfish and from the southern hemisphere. Additionally, a key to all the species of this genus is provided.

**Supplementary Information:**

The online version contains supplementary material available at 10.1007/s11230-024-10189-6.

## Introduction

Members of the family Ergasilidae Burmeister, 1835 occur globally as parasites of freshwater, brackish, and marine fishes. There are currently 30 accepted genera in the family (Hadfield, 2019; Walter & Boxshall, 2024a), of which three have been reported from African freshwater fishes, namely: *Ergasilus* von Nordmann, 1832; *Neoergasilus* Yin, 1956 (an invasive species); and *Paraergasilus* Markevich, 1937 (Oldewage & van As, 1988a, b; Berrouk et al., 2018, 2020; Boucenna et al., 2018; Avenant-Oldewage et al., 2023; Fikiye et al., 2023). During parasitological surveys in South Africa, ergasilids were collected from the gills of the North African catfish, known locally as the sharptooth catfish, *Clarias gariepinus* (Burchell), in the Great Fish River, Eastern Cape, South Africa. This fish species has been translocated to the Great Fish River as a result of the Inter-Basin Water Transfer scheme from the Gariep Dam (Free State, South Africa) (see Cambray & Jubb, 1977). Currently, catfish populations are established in the Great Fish River and serve as hosts to several parasite species (see Truter et al., 2023a). The morphology of the ergasilid species collected from the current study conformed to the characteristics of members in the genus *Neoergasilus*. Similar to other ergasilid copepods, members of *Neoergasilus* are found attached to the gills and fins of their hosts (Hayden & Rogers, 1998; Hudson & Bowen, 2002; Alekseev et al., 2021).

There are currently nine accepted species of *Neoergasilus* (Walter & Boxshall, 2024b) described from China (*N. longispinosus* Yin, 1956); India (*N. ferozepurensis* Kumari, Khera & Gupta, 1988; *N. indicus* Vankara & Chikkam, 2010; *N. kherai* Battish & Brar, 1989; and *N. notopteri* Kumari, Khera & Gupta, 1988); Korea (*N. angustus* Kim & Choi, 2003 and *N. bullatus* Kim & Choi, 2003); Russia (*N. squaliobarbi* (Dogiel & Akhmerov, 1952) (syn. *N. inflatus* see Smirnova, 1971)); and Taiwan (*N. japonicus* (Harada, 1930)). However, *N. japonicus* is the only species that has been reported from multiple continents including Africa (Berrouk et al., 2018; 2020; Boucenna et al., 2018; Avenant-Oldewage et al., 2023), Asia (Harada, 1930; Urawa et al., 1980; Kumari et al., 2009; HongWei et al., 2010; Nagasawa & Sato, 2016), Europe (Lescher-Moutoué, 1979; Mugridge et al., 1982; Beyer et al., 2005; Vainikka et al., 2009; Alfonso & Belmonte, 2010; Soylu & Soylu, 2012; Kuş & Soylu, 2013; Elsheikha & Beech, 2017; Ondračková et al., 2019; 2021; Kvach et al., 2021; 2023), North America (Hayden & Rogers, 1998; Hudson & Bowen, 2002; Suárez-Morales & Mercado-Salas, 2013; Truong & Bullard, 2021), and South America (Mendes Marques & Murrieta Morey, 2019).

Although the specimens from this study conform to the genus *Neoergasilus,* they differed morphologically from all other nine species in this genus. Limited genetic data are available for species of this genus. Currently, the only available sequences on GenBank are for *N. japonicus* for ribosomal RNA genes, 18S and 28S from the Czech Republic (Ondračková et al., 2019; Kvach et al., 2021), South Africa and Japan (Avenant-Oldewage et al., 2023); and the mitochondrial DNA gene, COI, from South Korea (Baek et al., 2016) and the United States of America (Vasquez et al., 2021 supplementary data). There is also a GenBank submission from South Korea for 18S and 28S gene regions, but it is not associated with any peer-reviewed article. This study, therefore, aims to characterise a parasitic copepod species morphologically and molecularly from the genus *Neoergasilus* not previously known to science as well as provide a key for taxon identification in this genus.

## Materials and methods

*Sampling*

**Fig. 1:**
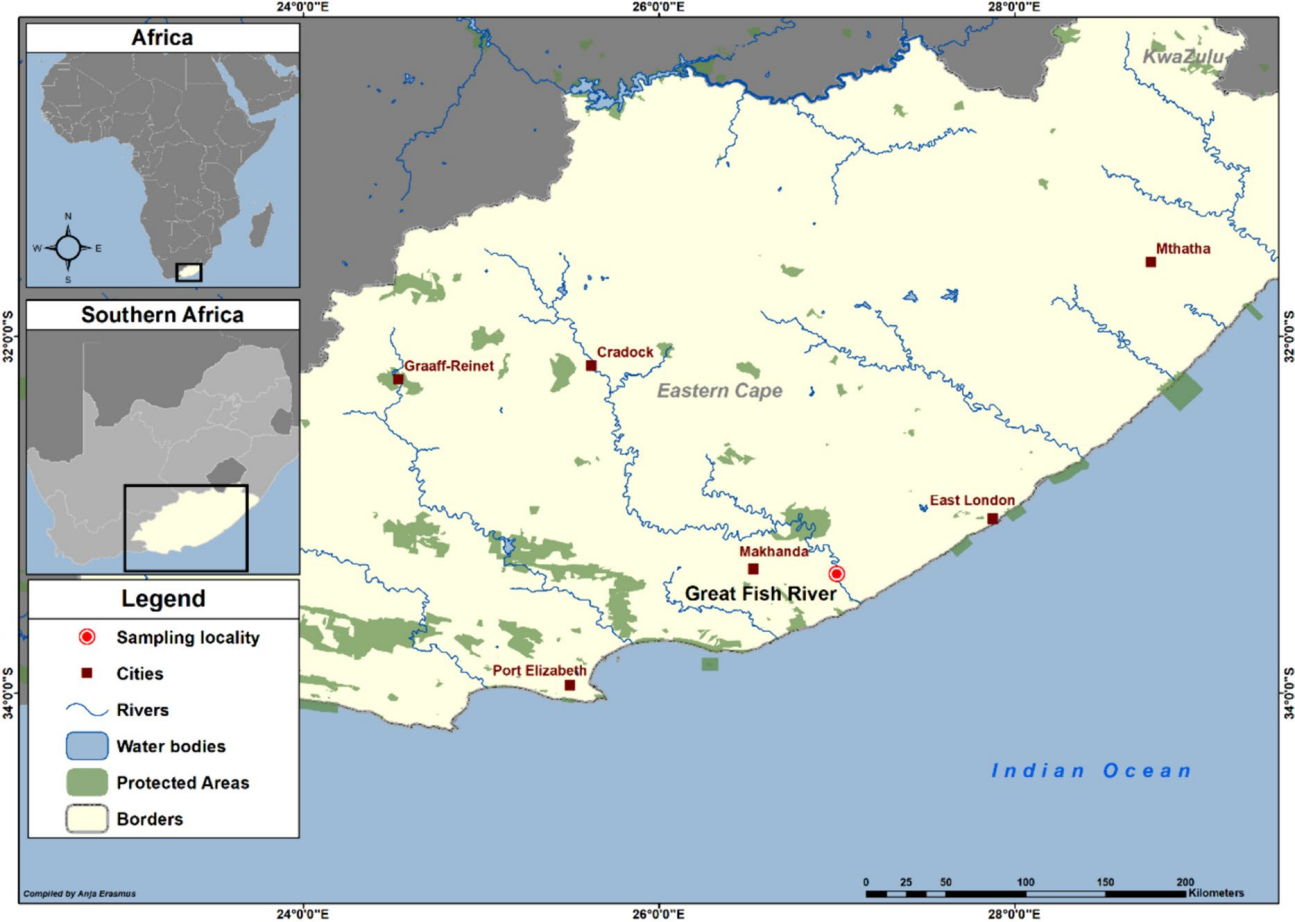
Map showing the sampling locality (Great Fish River, Eastern Cape, South Africa) for *Clarias gariepinus* (Burchell) in the Eastern Cape, South Africa

Fifteen specimens of *C. gariepinus* were caught with baited longlines in November 2018 from the Great Fish River, Eastern Cape, South Africa (33°19′49.3″S 26°59′54.2″E) (Fig. 1). Collected fish were examined for parasites attached to the body surface, fins, and gills, and were dissected and screened with the aid of a Zeiss Stemi 305 dissection microscope following standard methods for crustacean parasites as in Dávidová and Smit (2018). Adult female copepods (n = 21) were collected from the gills using fine paint brushes, needles, and tweezers, cleaned in Petri dishes containing freshwater, and fixed in 70% ethanol for morphological and molecular analyses. Host nomenclature is from FishBase (Froese & Pauly, 2024), host and parasite authorities (except for the synonymy) are not included in the references.

*Morphological analysis*

For morphology, photomicrographs of uncleared specimens were taken with the aid of a Zeiss Stemi 508 stereomicroscope (Zeiss, Oberkochen, Germany), then cleared in lactic acid for 60 minutes and stained in lignin pink for 10–20 minutes. With the aid of the stereomicroscope, six adult female specimens were dissected on glass microscope slides using fine needles, mounted temporarily with glycerine. Mounted specimens were viewed with bright-field and phase-contrast microscopy functions of a Nikon Eclipse 80i or Nikon Eclipse *Ni* differential interference contrast (DIC) microscopes (Nikon Instruments, Tokyo, Japan), enabling the z-dimensional stacking function. Photomicrographs of dissected appendages were taken using NIS-Elements BR Ver. 4.60 software of the Nikon microscopes and drawn with the aid of a drawing tube microscope attachment. Measurements of 10 randomly selected specimens were made using the Labscope Mat Ver. 2.8.4 microscope software connected to the Zeiss Stemi 508 stereomicroscope. All measurements are expressed in millimetres as mean ± standard deviation (with range in parentheses). Six ovigerous female specimens were selected for scanning electron microscopy (SEM). Specimens were dehydrated through a graded alcohol series; dried using hexamethyldisilazane (HMDS) or critically point dried; coated with gold-palladium and studied with the aid of a JEOL Winsem JSM IT 200 at 5 KVa. Illustrations were electronically inked using a Wacom Intuos® Pro drawing tablet and Adobe Illustrator™ software package. Morphological terminologies for the description followed Abdelhalim et al. (1993) and Boxshall (2016).

*Infestation rates*

Calculations for infestation rates were according to Bush et al. (1997), including prevalence, intensity, mean intensity, and mean abundance (± standard error).

*Molecular analysis*

Two protocols were used for genomic DNA extractions. The Macherey-Nagel tissue kit (GmbH & Co. KG, Sandton, South Africa) followed the manufacturer’s protocol with an adaptation of the pre-lysis incubation period (3 hours ± 30 minutes in shaking incubator and a hold time of 2 hours), was used to extract genomic DNA from an ovigerous female specimen. The PCRBIO Rapid Extract PCR Kit (PCRBiosystems, Analytical Solutions, Randburg, South Africa) was also used for the extraction of genomic DNA using one egg string each from three separate specimens that had been selected for dissection, adapting the manufacturer’s protocol as follows: 20 µl of lysis buffer, 10 µl of buffer containing protease, and 150 µl molecular grade water for dilution.

DNA amplifications for partial ribosomal RNA gene regions used primers designed by Song et al. (2008): 18SF and 18SR (18S), and 28SF and 28SR (28S); and the partial mitochondrial DNA gene region (COI) was amplified with primers by Bucklin et al. (2010): cop-Col-1498-F and cop-Col-2198-R (Table 1). Each Polymerase Chain Reaction (PCR) was carried out in 25 µl volumes using: 12.5 μl of DreamTaq PCR Master Mix (Thermo Fisher Scientific, South Africa); 1.25–2.5 μl of 10 μM of each primer (forward and reverse); and 2.5–10 μl of DNA product. The proportion of molecular grade water varied amongst the samples to make the final volume of 25 μl. DNA amplification was performed in a Benchmark TC9639 Thermal Cycler (Whitehead Scientific (Pty.) Ltd., Benchmark Scientific, USA) following conditions by Song et al. (2008) for 18S and adapting the conditions by Hayes et al. (2021) for 28S and COI (Table 1). Negative controls were used to detect possible contamination of the reagents.

**Table 1:** List of primers with references, annealing temperatures, and the number of cycles used for DNA amplification of *Neoergasilus africanus* **n. sp**. from the Great Fish River, South Africa

Gene regions	Specimen codes	GenBank accession numbers	Primers	Sequences	Annealing temperature (˚C)	Number of cycles	Primer references
18S	P40-R P47-K P59-R	PP864457 PP864458 PP864459	18SF	5′-AAG GTG TGM CCT ATC AAC T-3′	54	30	Song et al. (2008)
			18SR	5′-TTA CTT CCT CTA AAC GCT C-3′			
28S	P50-Ra2 P59-Ra2	PP864460 PP864461	28SF	5′-ACA ACT GTG ATG CCC TTA G-3′	47	40	
			28SR	5′-TGG TCC GTG TTT CAA GAC G-3′			
COI	P50-RJ P59-RJ	PP866728 PP866729	cop-Col-1498-F	5′-GGGTGACCAAAAAATCARAA-3'	47	40	Bucklin et al. (2010)
			cop-Col-2198-R	5′-AAYCATAAAGAYATYGGDAC-3'			

GenBank accession numbers are provided for respective isolates. Abbreviations in the specimen code: R indicates specimens extracted using the PCRBio rapid kit extraction method; K indicates specimens extracted using the Machery-Nachel tissue kit extraction method

Amplicons of PCR were verified by 1% agarose gel electrophoresis along with a 1kb DNA ladder (BioLabs Inc.). Positive PCR products were sent to a commercial sequencing company, Inqaba Biotechnical Industries (Pty) Ltd., Pretoria, South Africa, for purification and sequencing in both directions. Forward and reverse sequences received were checked for ambiguity, assembled, and edited using the bioinformatics software platform, Geneious Prime v. 2022.2.2 (Biomatters, Auckland, New Zealand; Kearse et al., 2012). Basic Local Alignment Search Tool (BLAST) (https://blast.ncbi.nlm.nih.gov/Blast.cgi) was used to check the similarity between newly generated sequences with submissions of *Neoergasilus* available on GenBank as well as other copepods from the family Ergasilidae.

Due to the paucity of sequences from species in this genus, published and unpublished *N*. *japonicus* sequences were used in the alignment for pairwise genetic distance calculations. However, only sequences associated with peer-reviewed publications from the family Ergasilidae were used for the species of other ergasilid genera and *Lernaea cyprinacea* Linnaeus, 1758 (Lernaeidae) was used as the outgroup in this study (see Supplementary Table S1). Pairwise genetic distance matrices, expressed as the percentage similarity of bases and the number of base pair differences, were estimated in Geneious Prime v 2022.2.1.

## Results

*Taxonomy*

Order: Cyclopoida Burmeister, 1834

Family: Ergasilidae von Nordmann, 1832

Genus: ***Neoergasilus*** **Yin, 1956**

*Restricted synonymy*

*Ergasilus* Harada, 1930: 71–76 [not *Ergasilus* von Nordmann, 1832].

*Neoergasilus* Yin, 1956: 245–246; 267.—Yamaguti, 1963: 37.—Kabata, 1979: 81, 83.—Lescher-Moutoué, 1979: 111.— Mugridge, Stallybrass, & Hollman, 1982: 533.—Kumari, Khera, & Gupta, 1988: 163.—Battish & Brar, 1989: 54–57.—Pu-ren & De-sheng, 1992: 1–3.—Kim & Choi, 2003: 71–83.—Vankara & Chikkam, 2010: 425–434.

*Type-species*

*Ergasilus japonicus* Harada, 1930 [now *Neoergasilus japonicus* (Harada, 1930)] by original designation.

*Generic remarks*

Individuals from the genus *Neoergasilus* are mainly characterised by the morphology of the first leg and antennae. The first leg is elongate, reaching fourth and fifth pedigerous somites in some specimens; the basis of the first leg has a triangular process at the posterior margin between the endopod and exopod; and an enlarged spatulate spine is present on the outer margin of the second exopodal segment of the first leg (Yin, 1956; Kim & Choi, 2003). In addition, the antennae of *Neoergasilus* are short, slender, and strongly curved, with a conspicuous spine (also referred to as a seta in Kim & Choi, 2003) present on the basal or second segment. The specimens from the current study conformed to the generic characteristics mentioned above.

***Neoergasilus africanus*** **n. sp.**

**Fig. 2:**
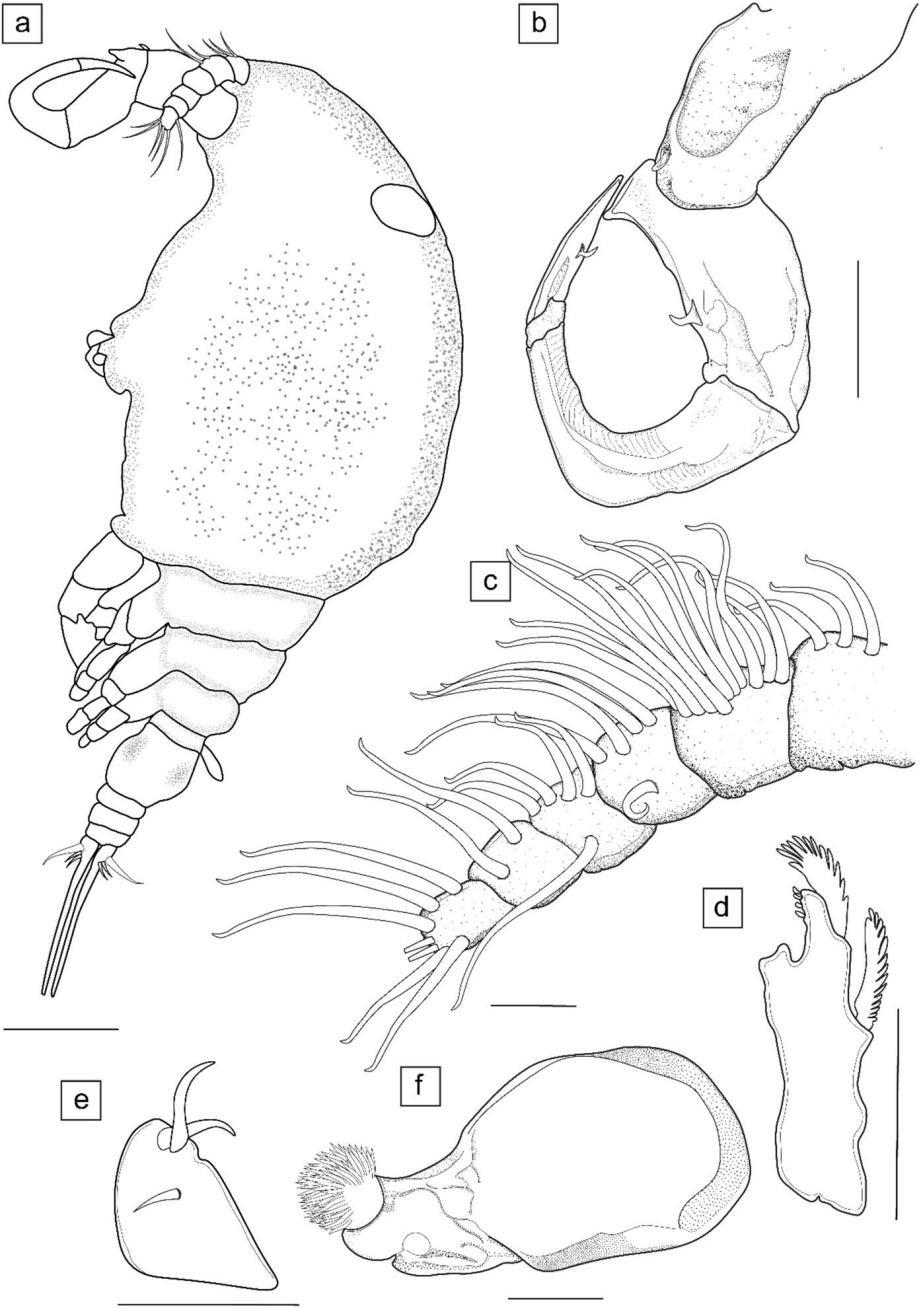
Illustrations of adult female *Neoergasilus africanus* **n. sp.** and the cephalic appendages. (**a**) lateral view, without eggs; (**b**) antenna; (**c**) antennule; (**d**) mandible; (**e**) maxillule; (**f**) maxilla. Scale bars: (**a**) 100 µm; (**b**,** e**) 50 µm; (**c**,** f**) 20 µm; (**d**) 25 µm

**Fig. 3:**
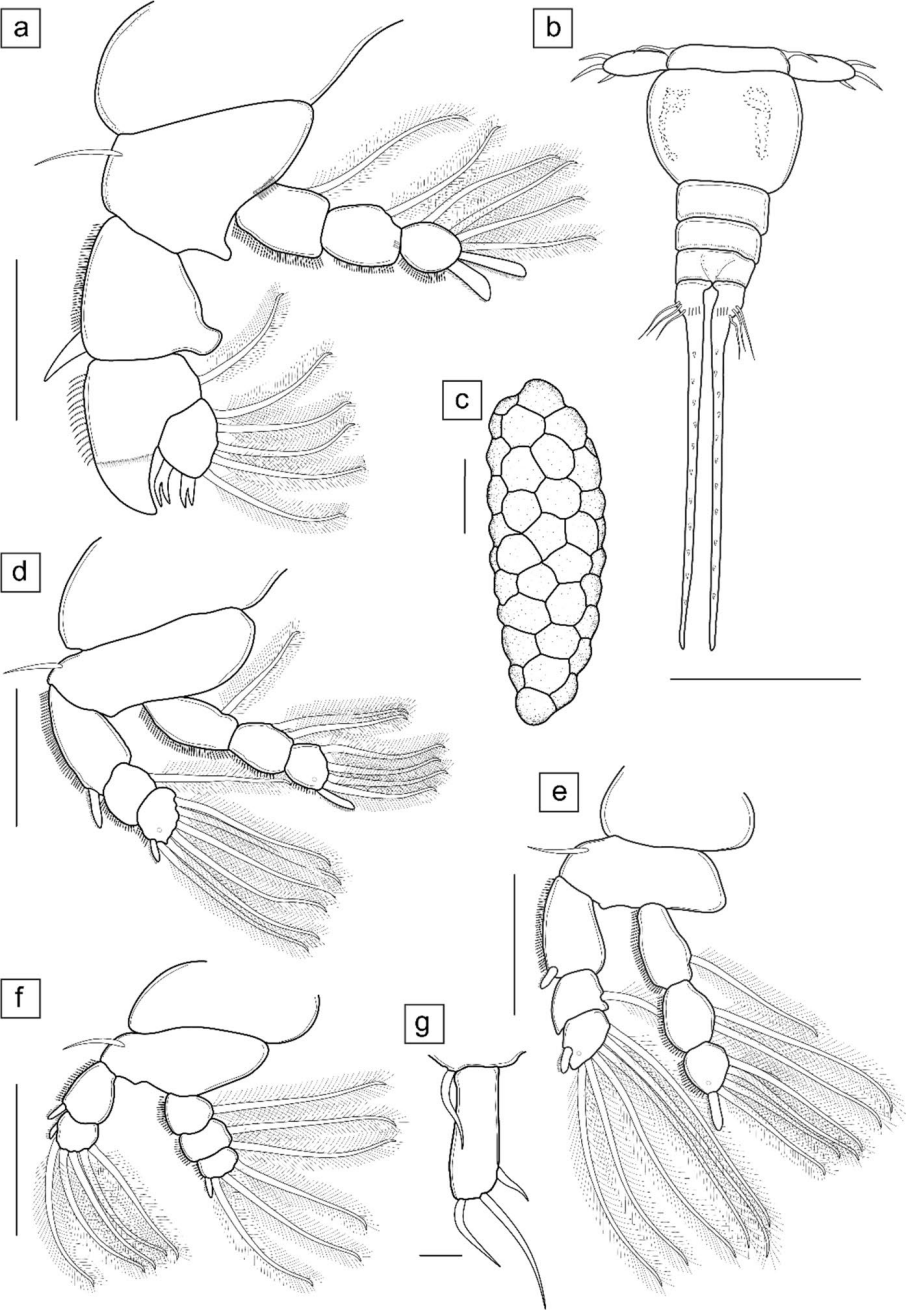
Illustrations of the legs, urosome, caudal rami and egg string of the adult female *Neoergasilus africanus* **n. sp.** (**a**) leg 1 showing triangular process on posterior margin of basis, knob-like process on inner distal margin of first exopodal segment, spatulate spine on outer distal margin of second exopodal segment extending longer than the two forked spines of third exopodal segment; (**b**) urosome showing ventral row of spinules on genital double-somite, abdominal somites, and caudal rami with simple setae of varying sizes, and median seta of caudal rami with an array of spines; (**c**) egg string; (**d**) leg 2; (**e**) leg 3; (**f**) leg 4; (**g**) leg 5. Scale bars: (**a**, **d–f**) 50 µm; (**b, c**) 100 µm; (**g**) 10 µm

**Fig. 4:**
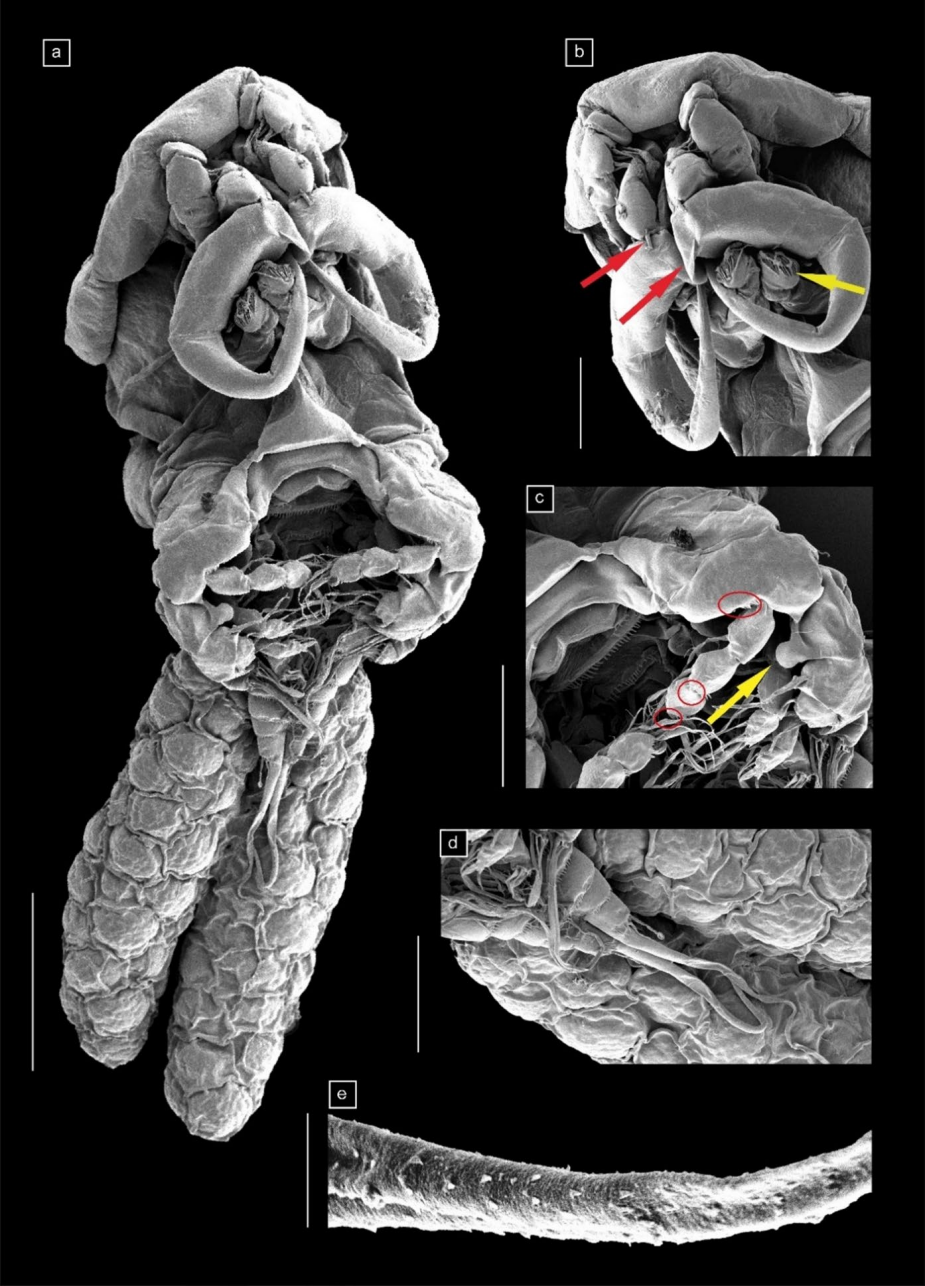
Scanning electron microscope (SEM) images of *Neoergasilus africanus* **n. sp.** adult female. (**a**) ventral view; with two egg strings; (**b**) ventral view of cephalosome showing spine and cone-like process of first and second antennal segments, respectively (red arrows), and maxilla (yellow arrow); (**c**) thoracic region showing denticles in segments of leg 1 (red circles) and knob-like process in inner margin of first exopodal segment of leg 1 (yellow arrow); (**d**) urosome; (**e**) median setae of caudal rami ornamented with an array of spines. Scale bars: (**a**) 100 µm; (**b–d**) 50 µm; (**e**) 5 µm. (Color figure online)

*Neoergasilus* sp. Truter, Hadfield & Smit, 2023a: 170–179. Figs. 2, 3, 4

*Material examined*

*Holotype*: Ovigerous female collected from the gills of *Clarias gariepinus* in the Great Fish River (33°19′49.3″S 26°59′54.2″E), Eastern Cape, South Africa, collected by M. Truter (Nov. 2018). Deposited in the collections of the National Museum, Bloemfontein, South Africa (NMB P-1040).

*Paratypes*: Five ovigerous females, same host and collection data as holotype. The specimens are deposited in the collections of the National Museum, Bloemfontein, South Africa (NMB P-1041).

*Other material*: 13 ovigerous females, same host and collection data as holotype (six used for SEM; six dissected; one female and three egg strings used for DNA extraction). The remaining specimens are in the possession of the Water Research Group at the North-West University (NWU) Potchefstroom, South Africa.

*Infestation rates for adult females*: Total prevalence 20% (3/15), intensity 1–13 (1, 7, 13); mean intensity 7 (21/3), mean abundance 1.40 (± 0.89).

*ZooBank registration number*: The Life Science Identifier (LSID) for this article is urn:lsid:zoobank.org:pub:78F4D442-AE4C-4DBD-B8BF-4E9EEC4C4610. The LSID for the new name *Neoergasilus africanus* **n. sp.** is urn:lsid:zoobank.org:act:A6185054-3B96-4A59-8CB9-7E5EA8A9CF0B.

*Representative DNA sequences*: Numbers of bases (bp) and GenBank accession numbers are given as follows:

18S: 1278, 1299, 1299 bp long sequences of three specimens, PP864457–PP864459

28S: 672, 697 bp long sequences of two specimens, PP864460–PP864461

COI: 655, 548 bp long sequences of two specimens, PP866728–PP866729.

*Etymology*: The species name *africanus* is chosen from the name of the continent “Africa” where the parasite was collected, as this is the first species from the genus *Neoergasilus* to be described from Africa. Additionally, the specific name is an adjective conforming to the masculine gender of the generic name.

*Description of adult female* (Figs. 2, 3, 4)

Measurements (n = 10) are given as: total length (from anterior of cephalothorax to posterior of caudal rami, excluding caudal rami seta) 0.70 ± 0.08 (0.54–0.78), cephalothorax length 0.41 ± 0.06 (0.28–0.48), cephalothorax width 0.30 ± 0.04 (0.21–0.36), urosome length 0.15 ± 0.02 (0.12–0.18).

Body cyclopiform (Figs. 2a, 4a), comprising prosome consisting of cephalosome and first pedigerous somite fused (=cephalothorax), with anterodorsal oval ornamentation present and three free pedigerous somites (bearing legs 2 to 4), and urosome consisting of fifth pedigerous somite, genital double-somite and three free abdominal somites. Cephalothorax greatly expanded, 0.75 times as wide as long. Distinct lateral constriction defining cephalothorax and second pedigerous somite. Second pedigerous somite distinctly separated from cephalothorax; third and fourth somites gradually reducing in size; fifth somite reduced in size and grouped as part of urosome. Genital double somite with genital opening, three free abdominal somites, and caudal rami all ornamented with vertical row of minute spinules, observed ventrally (Figs. 3b, 4d). Each abdominal somite overlapping, slightly larger than the next; anal somite with dorsoventral incision. Caudal ramus rectangular with four setae of varying lengths: median seta largest and longest, extending from the caudal ramus and ornamented with array of spines (Figs. 3b, 4e). Egg sacs (Fig. 3c) 0.80 times length of body, multiseriate, occurring in pairs, with each sac containing approximately three columns of 8–10 eggs.

Antennule (Fig. 2c) 6-segmented, with simple setae of varying lengths; setal armature formula (segments 1–6): 3, 11, 5, 5, 2, 5 + 2 aesthetascs; no aesthetascs seen on segments one to five. Antenna (Figs. 2b, 4b) 4-segmented; first segment 1.50 times as long as widest region, possesses small elongate spine on posterodistal margin (Fig. 2b); second segment 0.90 times as long as first; 0.58 times as wide as long, possesses cone-like basal process on proximal inner margin and short seta on post-medial inner margin of segment (Figs. 2b, 4b); third segment longest, 1.2 times as long as first segment, slender, tapering, strongly curved; fourth segment greatly reduced; short terminal claw about 0.6 times as long as first segment, armed with short seta at inner margin (Fig. 2b).

Mandible (Fig. 2d) biramous with three toothed blades: endopod comprising short anterior and long median blade; exopod represented as posterior blade. Maxillule (Fig. 2e) sightly elongate subtriangular lobe with two unequal setae and one small spine. Maxilla 2-segmented (Figs. 2f, 4b), distal segment armed with numerous teeth on anterior margin.

Swimming legs 1–4 (Figs. 3a, d–f) biramous, each with 3-segmented endopod and 3-segmented exopod, except for 2-segmented exopod of leg 4. Basis of legs 1–4 with simple spiniform setae on outer margins, all other setae on exo- and endopods plumose; outer margins of endo- and exopodal segments of legs 1–4 serrated; all spines of legs 1–4 serrated (except forked spines of third exopodal segment of leg 1). Leg 1 largest and longest, reaching up to fourth pedigerous somite; basis with distal horizontal row of 6–10 spines; second and third endopodal segments serrated at distal margins (Fig. 4c); knob-like process present on inner distal margin of first endopodal segment (Figs. 3a, 4c); spatulate spine on outer margin of second exopodal segment sharply pointed, projecting inwards, longer than third exopodal segment and spines (Fig. 3c). Legs 2 and 3 (Fig. 3d–e) with same spine-setae armature; single pore towards outer margin, on third segments of endopod and exopod of legs 2 and 3. Table 2 gives the armature formula of legs 1–4.

**Table 2:** Spine-setae formula for swimming legs of *Neoergasilus africanus* **n. sp.** from South Africa

	Coxa	Basis	Exopod	Endopod
Leg 1	0-0	0-1	I–0; I*–1; II–5	0–1; 0–1; II–4
Leg 2	0-0	0-1	I–0; 0–1; I–6	0–1; 0–2; I–4
Leg 3	0-0	0-1	I–0; 0–1; I–6	0–1; 0–2; I–4
Leg 4	0-0	0-1	I–0; I–5	0–1; 0–2; I–3

Number of spines in Roman numerals, number of setae in Arabic numerals. Spine with asterisks* represents the generic spatulate spine

Leg 5 (Fig. 3g) represented by a long seta at dorsolateral margin of fifth pedigerous somite, a free segment bearing three setae of unequal lengths; one sub-terminal seta (the shortest) and two longer terminal setae.

*Remarks*

*Neoergasilus africanus* **n. sp.** differs from the nine known species of *Neoergasilus* by a combination of the following characteristics: an inflated cephalothorax 0.75 times as wide as it is long; the presence of an oval dorsal ornamentation anterior to the cephalosome; a knob-like process on the inner distal margin of the first exopodal segment of leg 1 and two forked spines on the third exopodal segment of leg 1; leg 4 bearing a 2-segmented exopod and 3-segmented endopod; a single-segmented fifth leg with three distal unequal setae and a seta on the lateral margin of the fifth pedigerous somite; an elongate spine on the posterodistal margin of the first antennal segment, and cone-like basal process on proximal inner margin of its second antennal segment; the setation of the antennule; and the armature formula of the legs.

When compared to the other described *Neoergasilus* species, the inflated cephalosome of *N. africanus* **n. sp.** is similar to *N. squaliobarbi* and *N. bullatus*; the remaining seven species have a more elongate cephalosome. Furthermore, the anterodorsal oval ornamentation on the cephalosome is absent in *N. squaliobarbi* and *N. bullatus.* The stout knob-like process on the inner distal margin of the first exopodal segment of leg 1 observed in *N. africanus* **n. sp.** is absent in *N. squaliobarbi. Neoergasilus bullatus* possesses a long blade-like inner distal process, much longer than the segment itself compared to the knob-like process observed in *N. africanus* **n. sp.** Additionally, the two spines on the third exopodal segment of leg 1 are forked in *N. africanus* **n. sp.** but not forked in both *N. bullatus* and *N. squaliobarbi.* The antenna of *N. africanus* **n. sp.** has a cone-like process at the base of its second segment, which is absent in *N. bullatus* and *N. squaliobarbi*. The exopod and endopod of the fourth leg of *N. africanus* **n. sp.** are 2- and 3-segmented respectively, sharing similarities with *N. bullatus*, but differing from *N. squaliobarbi,* which has a single-segmented exopod and 2-segmented endopod. The fifth leg of *N. africanus* **n. sp.** is single-segmented, with a seta on the margin of the fifth pedigerous somite, similar to *N. bullatus.* The free segment of leg 5 in *N. africanus* **n. sp.** bears three unequal setae; *N. bullatus*, however, has only a long plumose terminal seta and a minute lateral seta. In the case of *N. squaliobarbi* and its synonym *N. inflatus*, the authors report an absent fifth leg.

Geographically, only *N. japonicus* has been reported in the southern hemisphere (Mendes Marques & Murrieta Morey, 2019; Berrouk et al., 2018; 2020; Avenant-Oldewage et al., 2023). The species described in this study differs from *N. japonicus* with several morphological characters. The cephalosome is inflated compared to the more elongate cephalosome of *N. japonicus*. *Neoergasilus africanus* **n. sp.** also has an anterodorsal oval ornamentation on the cephalothorax otherwise absent (or not observed) in the descriptions of *N. japonicus*. In addition to the elongate spine on the posterodistal margin of the first antennal segment, *N. africanus* **n. sp.** is ornamented with a basal cone-like process at the proximal margin of its second antennal segment, which is absent on the antennae of *N. japonicus.* The fourth leg of *N. africanus* **n. sp.** has a 2-segmented exopod and a 3-segmented endopod, whereas *N. japonicus* possesses a single-segmented endo- and exopod. Additionally, the spine-setae formulae of the legs are different for *N. africanus* **n. sp.** and *N. japonicus*.

*Key to the species of* Neoergasilus

**Fig. 5:**
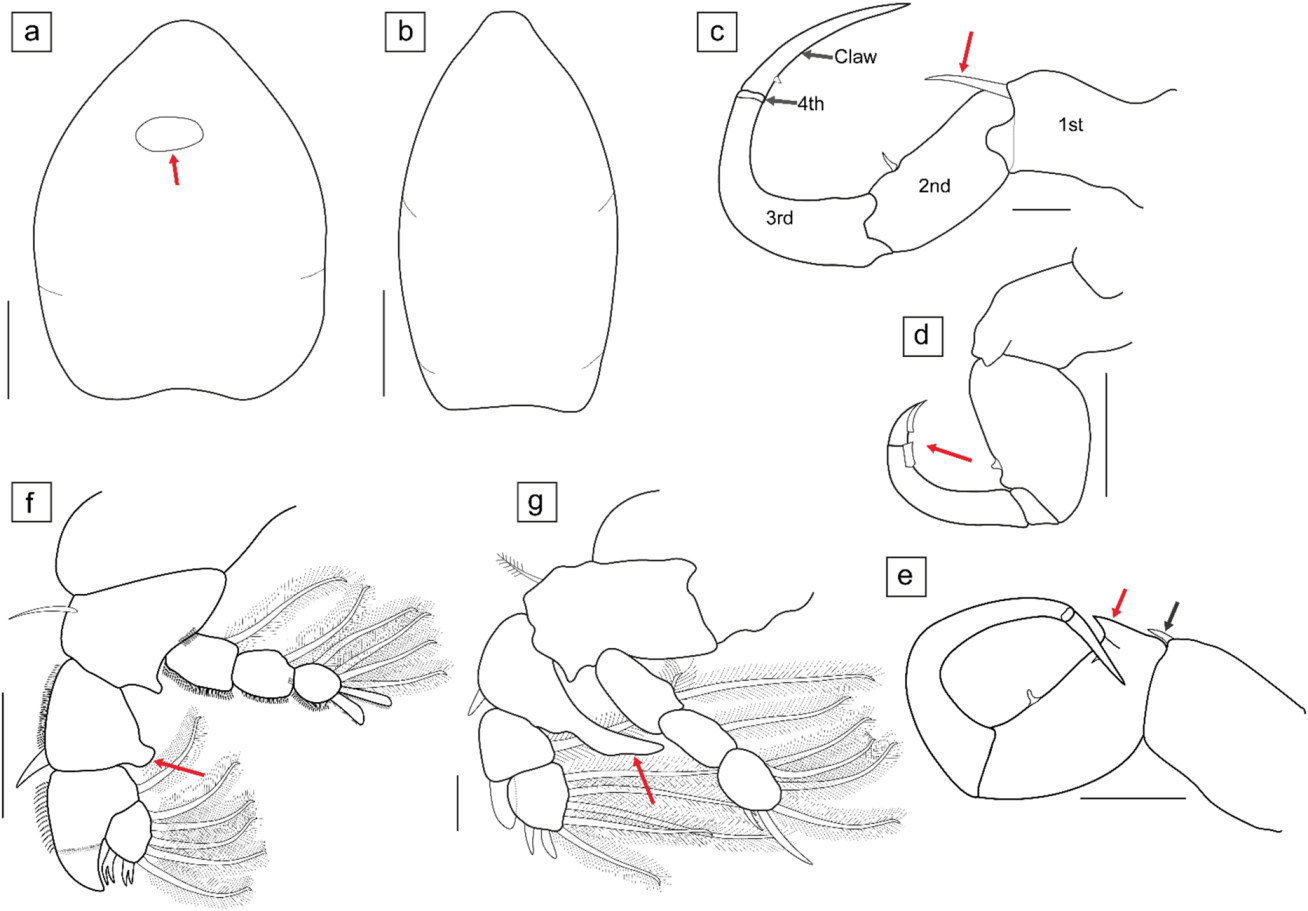
Illustrations of certain morphological characters used for keying out *Neoergasilus* Yin, 1956 species. (**a**) inflated cephalothorax with oval ornamentation (red arrow); (**b**) elongate cephalothorax without any ornamentation; (**c**) antenna showing the 1^st^, 2^nd^, 3^rd^, 4^th^ segments and the claw; with scalpel-like spine (red arrow); (**d**) antenna highlighting short and broad claw with tooth-like projection on inner margin (red arrow); (**e**) antenna with short elongate spine (black arrow) and a cone-like basal process (red arrow); (**f**) leg 1 with knob-like bulging process (red arrow) on inner margin of first exopodal segment; (**g**) leg 1 with blade-like process (red arrow) on inner margin of first exopodal segment. Scale bars: (**a**, **b**) 100 µm; (**c**, **g**) 20 µm; (**d–f**) 50 µm. (Color figure online)

To date, the only available key to the species of *Neoergasilus* was published by Kumari et al. (1988), comprising the five known species at the time, two of which were newly described by the authors. At that point, Smirnova (1971) had already synonymised *N. inflatus* with *N. squaliobarbi* (see Smirnova, 1971). Since then, four more species have been described and a fifth, *N. africanus* **n. sp.**, is described here. The key below was constructed using drawings and texts of original descriptions and supporting descriptions of the 10 known species. The main morphological characteristics used in the key are illustrated in Fig. 5.

This key is based on the morphological characters of the adult female:

1. Cephalothorax inflated (Fig. 5a) (at least 0.75 times as wide as long)……… 2
  Cephalothorax elongate (Fig. 5b) (less than 0.75 times as wide as long)……… 4
2. Antennal claw short and broad with tooth-like projection on inner margin (Fig. 5d); first exopodal segment of leg 1 with smooth inner margin………***N. squaliobarbi***
  Antennal claw without tooth-like projection on inner margin (Figs. 5c, e); first exopodal segment of leg 1 with bulging process on inner margin………3
3. Oval ornamentation on cephalothorax (Fig. 5a); knob-like process on inner margin of first exopodal segment of leg 1 (Fig. 5f); elongate spine on distal margin of first antennal segment with proximally placed cone-like process on second antennal segment………***N. africanus*** **n. sp.**
  Cephalothorax without oval ornamentation; blade-like process on inner margin of first exopodal segment of leg 1 (Fig. 5g); first antennal segment with one large, scalpel-like distal spine (Fig. 5c) ………***N. bullatus***
4. Leg 1 with bulging process on inner or outer margin of first exopodal segment………5
  Leg 1 without bulging process on inner or outer margin of first exopodal segment………7
5. Knob-like process present (Fig. 5f) on inner margin of first exopodal segment of leg 1………6
  Elongate blade-like process present (see Fig. 5g) on outer distal margin of first exopodal segment of leg 1; Leg 5 uniramous, single-segmented with two long terminal setae and one short lateral seta………***N. kherai***
6. Leg 5 uniramous, two-segmented with single seta on proximal segment and three unequal setae on distal segment……… ***N. indicus***
  Leg 5 uniramous, single-segmented with three setae; spines on outer margin of first exopodal segment less than half the length of second segment in legs 2–4……… ***N. notopteri***
7. Leg 4 with single-segmented exo- and endopod; first antennal segment with elongate distal spine (Fig. 5e); spines on outer margin of first exopodal segment less than half the length of second segment in legs 2–4……… ***N. japonicus***
  Leg 4 with at least 2-segmented endo- and exopod……… 8
8. Spines on outer margin of first exopodal segment longer than second segment in legs 2–4……… 9
  Spines on outer margin of first exopodal segment longer than third segment in legs 2–4; leg 5 with three equal setae……… ***N. longispinosus***
9. Leg 5 uniramous with two setae; second antennal segment with one large, scalpel-like spine (Fig. 5c) towards proximal margin……… ***N. ferozepurensis***
  Leg 5 with one dorsolateral seta on fifth pedigerous somite and three unequal setae on free segment; first antennal segment with one large, scalpel-like distal spine (Fig. 5c) ……… ***N. angustus***

The table of the characteristics used to compare all ten *Neoergasilus* species and to generate this key can be found in the appendix (Supplementary Table S2).

*Molecular analysis*

Seven novel sequences were generated from this study (three sequences for 18S, two sequences for 28S, and two sequences for COI). The phylogenetic alignments were done at family level since only one species from *Neoergasilus*, namely *N. japonicus*, has sequences available in GenBank (see Supplementary Table S1).

Phylogenetic alignments of the 18S sequences yielded an alignment length of 1326 bases. All newly generated sequences for *N. africanus* **n. sp.** were 100% identical. They had a similarity of 97.54–97.65% (30–32 bp differences) when compared to the *N. japonicus* sequences; 96.62–97.89% (21–44 bp) for *Ergasilus* species; 97.69–97.81% (28–30 bp) for *Sinergasilus* species; 96.95–97.00% (39 bp) for *Acusicola* species; 96.69–96.79% (41–43 bp) for *Paraergasilus* species; and 89.50–89.87% (132–139 bp) for *Lernaea* species (see Supplementary Table S3).

The phylogenetic alignment of the partial 28S gene region resulted in a length of 757 bases. The newly generated *N. africanus* **n. sp.** sequences were 100% identical. Compared to the *N. japonicus* sequences, they showed a 90.28–91.08% similarity (58–62 bp differences) (see Supplementary Table S4). All newly generated sequences for *N. africanus* **n. sp.** sequences had a similarity of 88.76–94.06% (40–78 bp) when compared to the *Ergasilus* species; 87.67–90.58% (62–85 bp) for *Sinergasilus* species; 91.73–91.76% (53 bp) for *Acusicola* species; 91.68–92.56% (50–55 bp) for *Paraergasilus* species; 70.24% (211 bp) for *Lernaea* species (see Supplementary Table S4).

No phylogenetic alignments were done for the COI gene due to the limited number of sequences available, however, a preliminary alignment suggested that the primers used for DNA amplification of *N. africanus* **n. sp.** amplified a fragment of this gene gene that is different from the fragments amplified for the other *Neoergasilus* and Ergasilidae sequences available on GenBank. The COI sequences are therefore provided for future studies of this genus and family.

## Discussion

The parasites collected from this study are placed in the genus *Neoergasilus* based on the morphology of the first swimming leg and features of the antennae. The genus *Neoergasilus* was originally described from Asia by Yin (1956) who described two new species, *N. inflatus* and *N. longispinosus. Neoergasilus inflatus* has since been synonymised with *N. squaliobarbi* (see Smirnova, 1971) because of the inflated cephalosome and teeth on the inner margin of the antennal claw (Fig. 5d). However, it appears that the synonymising of *N. inflatus* is not recognised by all authors or the status may still be unknown, as some authors still refer to *N. inflatus* as a recognised species (see Kim & Choi, 2003). The invasive species, *N. japonicus* is the only species from this genus that has been reported from Africa (Berrouk et al., 2018; 2020; Boucenna et al., 2018; Avenant-Oldewage et al., 2023). The specimens from the current study, morphologically differ from *N. japonicus* as well as the other eight congeners by a combination of features described in this paper.

Within the genus *Neoergasilus*, certain morphological characters have been described differently by some authors. The antennae, for instance, which is 4-segmented (Abdelhalim et al., 1993; El-Rashidy & Boxshall, 2002; Boxshall, 2016), is referred to as the second antenna by earlier authors and reported as 5-segmented by others. The description of the Ergasilidae antennae as given by El-Rashidy and Boxshall (2002) is 4-segmented (coxobasis and three endopodal segments) with a claw (see Fig. 5c). The first antennal segment is the coxobasis, and the second to fourth antennal segments are the first to third endopodal segments (see antennal segments in Fig. 5c). It may be assumed that some authors have included the antennal claw as a segment (resulting in a 5-segmented antenna), rather than as an attachment to the third endopodal segment. Kumari et al. (1988), for instance, refer to the coxobasis as the first and second segments, and the terminal claw is referred to as the fifth segment. Also, Kim and Choi (2003), in their description of *N. japonicus*, omitted the fourth antennal segment, which is visible from their drawing and referred to the claw as the fourth segment. This might be because the fourth segment is greatly reduced and might easily be missed if not consciously inspected.

Furthermore, many of the earlier authors had either described the antennal spine to be on the second segment (Harada, 1930; Dogel’ & Akhmerov, 1952; Vankara & Chikkam, 2010), at the margin between the second and third segment (Battish & Brar, 1989), or on the margin of the third segment (Kumari et al., 1988). It is also noteworthy that two characteristic structures of the antennae have been reported on the first and second (or second and third according to other earlier authors) antennal segments. The first is an elongate spine on the first segment (see red arrow in Fig. 5c and black arrow in Fig. 5e), which is usually at the distal margin of the segment and may also vary in length according to species; and the second is a slightly broader cone-like process, which is stout and pointed, almost extending from the base of the second segment (see red arrow in Fig. 5e). Four *Neoergasilus* species are described as having a slender spine on the first segment (*N. angustus*, *N. bullatus*, *N. japonicus*, *N. longispinosus* (see Yin, 1956; Kim & Choi, 2003)), while *N. indicus* and *N*. *notopteri* are described as having a cone-like process. *Neoergasilus ferozepurensis* is also described with a slender spine, and from the drawing, the spine is placed on the base of the second segment. For *N. squaliobarbi* (syn. *N. inflatus*) only a swollen disc-shaped process is reported on the second antennal segment (Dogel’ & Akhmerov, 1952). The antennae of *Neoergasilus* species may also be armed with other non-characteristic setae, spines, and papillae along the inner and outer margins of different segments. *Neoergasilus africanus* **n. sp.**, has two characteristic structures: the elongate spine on the first antennal segment, similar to *N. angustus*, *N. bullatus*, *N. japonicus*, and *N. longispinosus*; and a sharply pointed cone-like process extending from the basal end of the second antennal segment similar to *N*. *notopteri* and *N. indicus.*

Due to the limited sequences from this genus, an evolutionary relationship could not be estimated. Preliminary trees for 18S and 28S, however, support the position of this species as a member of the family Ergasilidae.

## Conclusion

The specimens from this study are placed in the genus *Neoergasilus* based on the morphology of the first legs and antennae, they differ from the nine known species by a combination of morphological characteristics and have thus been described as new to science. These specimens were collected from the gills of the catfish *C. gariepinus* sampled from the Great Fish River in the Eastern Cape Province, South Africa. This is the first species from this genus to be described from Africa and the southern hemisphere and is the first *Neoergasilus* species to be described from this host. The only previous records of ergasilids from this catfish species include *Ergasilus lamellifer* Fryer, 1961, *E. lizae* Krøyer, 1863, *E. mirabilis* Oldewage & van As, 1987, and *E. sarsi* Capart, 1944 (see Fikiye et al., 2023; Truter et al., 2023b). Apart from *N. japonicus*, there are no other *Neoergasilus* species with genetic data to explore the relationship of this new species in the genus. There is, therefore, a need for more genetic data to be provided with species description records in order to further understand the evolutionary relationship that exists within the genus *Neoergasilus* and the family Ergasilidae.

## Supplementary Information

Below is the link to the electronic supplementary material.

Supplementary file1 (TIF 221632 KB)

Supplementary file2 (TIF 208059 KB)

Supplementary file3 (DOCX 28 KB)

Supplementary file4 (DOCX 18 KB)

Supplementary file5 (XLSX 18 KB)

Supplementary file6 (XLSX 20 KB)

## Data Availability

All sequences generated from this study have been submitted in the GenBank database under the following Accession numbers PP864457-PP864459 (for 18S), PP864460-PP864461 (for 28S), and PP866728–PP866729 (for COI). The Holotype and paratypes from this study have been deposited in the collections of the National Museum, Bloemfontein, South Africa (NMB: P-1040 and NMB: P-1041). All other datasets generated during and/or analysed during the current study are available from the corresponding author upon reasonable request.
