# Supplementary material for: A new species of Neoergasilus Yin 1956 (Copepoda: Cyclopoida: Ergasilidae) parasitic on the catfish Clarias gariepinus (Burchell, 1822) (Siluriformes: Clariidae) from South Africa
Source: Syst Parasitol. 2024 Sep 24;101(5):64. doi: 10.1007/s11230-024-10189-6 (PMC11422265; doi:10.1007/s11230-024-10189-6)
Supplement: Supplementary file 3 — Supplementary file3 (DOCX 28 KB) [file 11230_2024_10189_MOESM3_ESM.docx]

**Supplementary Table S1** List of sequences included in the alignment for pairwise genetic distances. The taxa in bold are newly generated sequences, all other sequences are from GenBank and the Barcode of Life Database (BOLD). Site locality is only given for newly generated sequences. *Lernaea cyprinacea* Linnaeus, 1758 (in grey shade) was used as the outgroup

|  |  |  | **GenBank Accession Numbers** | | |  |
| --- | --- | --- | --- | --- | --- | --- |
| **Taxon** | **Host** | **Country** | **18S** | **28S** | **COI** | **Reference** |
| *Acusicola margulisae* | *Amphilophus citrinellus*, *Parachromis managuensis*, *Oreochromis* sp.; *Poecilia mexicana* | Nicaragua | MN852694 | MN852851 | MN854870 | Santacruz et al. (2020) |
| *Ergasilus anchoratus* | *Pseudobagrus fulvidraco* | China | DQ107564 | DQ107528 | - | Song et al. (2008) |
| *Ergasilus briani* | *Misgurnus anguillicaudatus* | China | DQ107572 | DQ107532 | - |  |
| *Ergasilus caparti* | *Neolamprologus brichardi* | Burundi | OQ407469 | OQ407474 | - | Míč et al. (2023) |
| *Ergasilus hypomesi* | *Acanthogobius hasta* | China | DQ107573 | DQ107539 | - | Song et al. (2008) |
| *Ergasilus* *macrodactylus* | *Gnathochromis permaxillaris* | Burundi | OQ407465 | OQ407470 | - | Míč et al. (2023) |
| *Ergasilus megacheir* | *Simochromis diagramma* | Burundi | OQ407466 | OQ407471 | - | Míč et al. (2023) |
| *Ergasilus parasarsi* | *Simochromis diagramma* | Burundi | OQ407467 | OQ407473 | - | Míč et al. (2023) |
| *Ergasilus parvus* | *Spathodus erythrodon* | Burundi | OQ407468 | OQ407472 | - | Míč et al. (2023) |
| **Ergasilus parasiluri* | *Tachysurus fulvidraco* | China | DQ107567 | DQ107536 | - | Song et al. (2008) |
| *Ergasilus peregrinus* | *Siniperca chuatsi* | China | DQ107577 | DQ107531 | - | Song et al. (2008) |
| *Ergasilus scalaris* | *Tachysurus dumerili* | China | DQ107565 | DQ107538 | - | Song et al. (2008) |
| *Ergasilus sieboldi* | *Perca fluviatilis* | Czech Republic | MW810238 | MW810242 | - | Kvach et al. (2021) |
| *Ergasilus* sp. | Free-living | South Korea | - | - | KR049035 | Baek et al. (2016) |
| *Ergasilus* sp. | *Mugil liza* | Argentina | - | - | KU557411 | Castro-Romero et al. (2016) |
| *Ergasilus tumidus* | *Acanthorhodeus taenianalis* | China | DQ107569 | DQ107535 | - | Song et al. (2008) |
| *Ergasilus wilsoni* | Free-living | South Korea | - | - | KR049036 | Baek et al. (2016) |
| *Ergasilus yaluzangbus* | *Gymnocypris stewartii* | China | DQ107578 | DQ107540 | - | Song et al. (2008) |
| **Ergasilus yandemontei* | *Odontesthes hatcheri* | Argentina | MT969345 | - | - | Waicheim et al. (2021) |
| ***Neoergasilus africanus* n. sp.** | ***Clarias gariepinus*** | **Great Fish River, South Africa** | **PP864457-PP864459** | **PP864460-PP864461** | **PP866728–PP866729** | **Present study** |
| *Neoergasilus japonicus* | *Lepomis gibbosus* | Czech Republic | MH167969 | MH167967 | - | Ondračková et al. (2019) |
| *Neoergasilus japonicus* | *Lepomis gibbosus* | Czech Republic | MH167970 | MH167968 | - | Ondračková et al. (2019) |
| *Neoergasilus japonicus* | *Lepomis gibbosus* | Czech Republic | MW810236 | MW810240 | - | Kvach et al. (2021) |
| *Neoergasilus japonicus* | *Lepomis gibbosus, Scardinos erythrophthalmus* | Czech Republic | MW810237 | MW810241 | - |  |
| *Neoergasilus japonicus* | Collected by plankton net | USA | - | - | MZ964933–MZ964936 | Vasquez et al. (2021) |
| *Neoergasilus japonicus* | Free-living | South Korea | - | - | KR049037 | Baek et al. (2016) |
| ***Neoergasilus japonicus* | *Notropis heterolepis* | USA | - | - | ZOOPS113-18 | BOLD (2023) |
| *Neoergasilus japonicus* | *Lepomis macrochirus* | Japan | OR350594 | OR347574 | - | Avenant-Oldewage et al. (2023) |
| *Neoergasilus japonicus* |  |  | OR350595 | OR347575 | - |  |
| *Neoergasilus japonicus* |  |  | - | OR347576 | - |  |
| *Neoergasilus japonicus* | *Labeobarbus cf. marequensis* | South Africa | OR350596 | OR347577 | - |  |
| *Neoergasilus japonicus* | *Enteromius cf. paludinosus* |  | OR350597 | OR347578 | - |  |
| *Neoergasilus japonicus* | *Labeobarbus aeneus* |  | OR350598 | OR347579 | - |  |
| *Neoergasilus japonicus* |  |  |  | OR347580 | - |  |
| *Neoergasilus japonicus* | *Tilapia sparrmanii* |  | OR350599 | OR347581 | - |  |
| *Neoergasilus japonicus* |  |  | OR350600 | OR347582 | - |  |
| *Neoergasilus japonicus* | Not given | South Korea | KR048752 | KR048823 | - | Baek et al. (Unpublished) |
| *Paraergasilus brevidigitus* | *Cyprinus carpio* | China | DQ107576 | DQ107530 | - | Song et al. (2008) |
| *Paraergasilus longidigitus* | *Abramis brama, Perca fluviatilis, Scardinius erythrophthalmu* | Czech Republic | MW810239 | MW810243 | - | Kvach et al. (2021) |
| *Paraergasilus medius* | *Ctenopharyngodon idellus* | China | DQ107574 | DQ107529 | - | Song et al. (2008) |
| *Sinergasilus major* | *Ctenopharyngodon idella* | China | DQ107560 | DQ107524 | - |  |
| *Sinergasilus major* | *Silurus glanis* | Hungary | MZ047814 | MZ047815 | - | Dos Santos et al. (2021) |
| *Sinergasilus polycolpus* | *Hypophthalmichthys molitrix* | China | DQ107563 | DQ107525 | - | Song et al. (2008) |
| *Sinergasilus polycolpus* | *Hypophthalmichthys molitrix* | China | - | - | KR263117 | Feng et al. (2016) |
| *Sinergasilus undulatus* | *Cyprinus carpio* | China | DQ107561 | DQ107526 | - | Song et al. (2008) |
| *Sinergasilus undulatus* | *Cyprinus carpio* | China | - | - | MW080644 | Hua et al. (2021) |
| *Lernaea cyprinacea* | *Carassius auratus, Cyprinus carpio, Chanodichthys ilishaeformis* | China | MH982195 | MH982204 | MH982220 | Hua et al. (2019) |

* *Ergasilus yandemontei* (Published on GenBank as *Ergasilus* sp.); *Ergasilus parasiluri* (published on GenBank as its synonym *Pseudergasilus parasiluri*).

** taxon from the Barcode of Life Database (BOLD, 2023).

**References to Supplementary Table S1**

Avenant-Oldewage, A., Nagasawa, K., Dos Santos, Q. M., & Oldewage, W. H. (2023). Pathology caused by introduced *Neoergasilus japonicus* (Copepoda: Ergasilidae) to the skin of indigenous *Tilapia sparrmanii* in South Africa and scanning electron microscopy study of wound-inflicting structures. *Journal of Fish Diseases*, 1–10. <https://doi.org/10.1111/jfd.13867>

Baek, S. Y., Jang, K. H., Choi, E. H., Ryu, S. H., Kim, S. K., Lee, J. H., Lim, Y. J., Lee, J., Jun, J., Kwak, M., Lee, Y. S., Hwang, J. S., Venmathi Maran, B. A., Chang, C. Y., Kim, I. H., & Hwang, U. W. (2016). DNA Barcoding of metazoan zooplankton copepods from South Korea. *PLoS ONE*, *11*(7), 1–20. <https://doi.org/10.1371/journal.pone.0157307>

BOLD, Barcode of Life Database (2023). *Neoergasilus japonicus* specimen record. Date accessed: 25th February 2023. Available at: <https://www.boldsystems.org/index.php/Public_RecordView?processid=ZOOPS113-18>

Castro-Romero, R., Montes, M. M., Martorelli, S. R., Sepulveda, D., Tapia, S., & Martínez-Aquino, A. (2016). Integrative taxonomy *of Peniculus, Metapeniculus,* and *Trifur* (Siphonostomatoida: Pennellidae), copepod parasites of marine fishes from Chile: species delimitation analyses using DNA barcoding and morphological evidence. *Systematics and Biodiversity*, *14*(5), 466–483. <https://doi.org/10.1080/14772000.2016.1158213>

Dos Santos, Q. M., Avenant-Oldewage, A., Piasecki, W., Molnar, K., Sellyei, B., & Szekely, C. (2021). An alien parasite affects local fauna-confirmation of *Sinergasilus major* (Copepoda: Ergasilidae) switching hosts and infecting native *Silurus glanis* (Actinopterygii: Siluridae) in Hungary. *International Journal for Parasitology: Parasites and Wildlife, 15*, 127–131. <https://doi.org/10.1016/j.ijppaw.2021.04.011>

Feng, H. L., Wang, L. X., Huang, J., Jiang, J., Tang, D., Fang, R., & Su, Y. B. (2016). Complete mitochondrial genome of *Sinergasilus polycolpus* (Copepoda: Poecilostomatoida). *Mitochondrial DNA. Part A*, *27*(4), 2960–2962. <https://doi.org/10.3109/19401736.2015.1060460>

Hua, C. J., Su, M. Y., Sun, Z. W., Lu, Y. H., & Feng, J. M. (2021). Complete mitochondrial genome of the copepod *Sinergasilus undulates* (Copepoda: Poecilostomatoida). *Mitochondrial DNA. Part B, Resources*,*6*(3), 1226–1228. <https://doi.org/10.1080/23802359.2020.1870890>

Hua, C. J., Zhang, D., Zou, H., Li, M., Jakovlic, I., Wu, S. G., Wang, G. T., & Li, W. X. (2019). Morphology is not a reliable taxonomic tool for the genus *Lernaea*: Molecular data and experimental infection reveal that *L. cyprinacea* and *L. cruciata* are conspecific. *Parasites and Vectors*, *12*(1), 579–591. <https://doi.org/10.1186/s13071-019-3831-y>

Kvach, Y., Tkachenko, M. Y., Seifertová, M., & Ondračková, M. (2021). Insights into the diversity, distribution, and phylogeny of three ergasilid copepods (Hexanauplia: Ergasilidae) in lentic water bodies of the Morava River basin, Czech Republic. *Limnologica*, *91*, 1–8. <https://doi.org/10.1016/j.limno.2021.125922>

Míč, R., Řehulková, E., & Seifertová, M. (2023). Species of *Ergasilus* von Nordmann, 1832 (Copepoda: Ergasilidae) from cichlid fishes in Lake Tanganyika. *Parasitology*, 1–72. <https://doi.org/10.1017/s0031182023000239>

Ondračková, M., Fojtů, J., Seifertová, M., Kvach, Y., & Jurajda, P. (2019). Non-native parasitic copepod *Neoergasilus japonicus* (Harada, 1930) utilizes non-native fish host *Lepomis gibbosus* (L.) in the floodplain of the river Dyje (Danube basin). *Parasitology Research*, *118*(1), 57–62. <https://doi.org/10.1007/s00436-018-6114-1>

Santacruz, A., Morales-Serna, F. N., Leal-Cardín, M., Barluenga, M., & Pérez-Ponce de León, G. (2020). *Acusicola margulisae* n. sp. (Copepoda: Ergasilidae) from freshwater fishes in a Nicaraguan crater lake based on morphological and molecular evidence. *Systematic Parasitology*, *97*(2), 165–177. <https://doi.org/10.1007/s11230-020-09906-8>

Song, Y., Wang, G. T., Yao, W. J., Gao, Q., & Nie, P. (2008). Phylogeny of freshwater parasitic copepods in the Ergasilidae (Copepoda: Poecilostomatoida) based on 18S and 28S rDNA sequences. *Parasitology Research*, *102*(2), 299–306. <https://doi.org/10.1007/s00436-007-0764-8>

Waicheim, M. A., Mendes Marques, T., Rauque, C. A., & Viozzi, G. (2021). New species of *Ergasilus* von Nordmann, 1832 (Copepoda: Ergasilidae) from the gills of freshwater fishes in Patagonia, Argentina. *Systematic Parasitology*, *98*(2), 131–139. <https://doi.org/10.1007/s11230-021-09966-4>
