# Supplementary material for: A new species of Neoergasilus Yin 1956 (Copepoda: Cyclopoida: Ergasilidae) parasitic on the catfish Clarias gariepinus (Burchell, 1822) (Siluriformes: Clariidae) from South Africa
Source: Syst Parasitol. 2024 Sep 24;101(5):64. doi: 10.1007/s11230-024-10189-6 (PMC11422265; doi:10.1007/s11230-024-10189-6)
Supplement: Supplementary file 4 — Supplementary file4 (DOCX 18 KB) [file 11230_2024_10189_MOESM4_ESM.docx]

**Supplementary Table S1:** Adult female morphological characters of the ten *Neoergasilus* species used in generating the new taxonomic key.

| **Characters** | ***Neoergasilus africanus* n. sp.** | ***Neoergasilus bullatus* Kim I.H. & Choi, 2003** | ***Neoergasilus squaliobarbi* (Dogiel & Akhmerov, 1952)**  **(syn. *Neoergasilus inflatus* Yin, 1956)** | ***Neoergasilus kherai* Batish & Brar, 1989** | ***Neoergasilus indicus* Vankara & Chikkam, 2010** |
| --- | --- | --- | --- | --- | --- |
| **Shape / size of cephalothorax** | inflated | inflated | inflated | elongate | elongate |
| **Spines / setae on antennae** | elongate spine on the proximal margin of first segment; stout cone-shaped spine emerging from the base of second segment | one large, scalpel-like inner distal seta; second segment with one papillate seta on distal third of inner margin; terminal claw with one proximal seta | basal segment does not have a disk of visible expansion, and there is a spine on the inner side of the antennae. | antenna basal segment devoid of any armature, second segment bears a small spine near the joint between the 2nd and 3rd segment; third segment longest and bears a long spine at some distance from the base | antenna basal segment naked; second segment with cone-like basal process and a small median spine; third segment sub-chelate |
| **Ornamentation on the cephalothorax** | one oval structure present | absent / not mentioned | absent / not mentioned | absent / not mentioned | absent / not mentioned |
| **Leg 1: processes on first exopodite** | knob-like process on inner distal margin | elongate blade process on inner distal margin | absent / not mentioned | elongate blade process on outer distal margin | knob-like process on inner distal margin |
| **Leg 4: exopod and endopod** | 2-segmented exopod and 3-segmented endopod | 2-segmented exopod and 3-segmented endopod | 1-segmented exopod and 2-segmented endopod | 2-segmented exopod and 3-segmented endopod | 2-segmented exopod and 3-segmented endopod |
| **Legs 2–4: length of first exopodal spines on outer margins** | less than half of second segment | less than half of second segment | less than half of second segment | less than half of second segment | less than half of second segment |
| **Leg 5: unsegmented or one-segmented** | uniramous, single segmented | uniramous, single segmented | not mentioned | uniramous, single segmented | uniramous, two segmented |
| **Leg 5: setation** | one seta extending from fifth pedigerous somite and three unequal setae attached to free segment | one dorsolateral seta of fifth pedigerous somite, free segment with one minute inner and one large, distally plumose terminal seta | not mentioned (for *N. inflatus*, not observed in Yin (1956), absent in Kim and Choi (2003)) | two long terminal setae, one short lateral seta | one short seta on proximal segment, three unequal setae on distal segment |

**Supplementary Table S1 contd.**

| **Characters** | ***Neoergasilus ferozepurensis* Kumari, Khera & Gupta, 1988** | ***Neoergasilus notopteri* Kumari, Khera & Gupta, 1988** | ***Neoergasilus angustus* Kim I.H. & Choi, 2003** | ***Neoergasilus longispinosus* Yin, 1956** | ***Neoergasilus japonicus* (Harada 1930)** |
| --- | --- | --- | --- | --- | --- |
| **Shape / size of cephalothorax** | elongate | elongate | elongate | elongate | elongate |
| **Spines / setae on antennae** | third segment bears a long spine at base | third segment curved around centre, forming a blunt short spine at base | first segment with one large, scalpel-like distal seta; second segment with one small seta at distal 1/4 of inner margin; terminal claw with one small proximal seta | long thorn at the junction of the distal inner side of the first segment and the third segment | first segment with a spine at its distal end, second segment with a minute spine at almost the middle of the inner border. |
| **Ornamentation on the cephalothorax** | absent / not mentioned | absent / not mentioned | one oval structure present (drawing) | absent / not mentioned | Absent / not mentioned (except drawing of Kim & Choi, 2003) |
| **Leg 1: processes on first exopodite** | absent / not mentioned | Knob-like process on inner distal margin | absent / not mentioned | absent / not mentioned | absent / not mentioned |
| **Leg 4: exopod and endopod** | 2-segmented endopod and exopod | 2-segmented exopod and 3-segmented endopod | 2-segmented endopod and exopod | 2-segmented exopod and 3-segmented endopod | 1-segmented exopod and endopod |
| **Legs 2–4: length of first exopodal spines on outer margins** | longer than second segment | less than half of second segment | longer than second segment | longer than third segment | less than half of second segment |
| **Leg 5: unsegmented or one-segmented** | uniramous, single segmented | uniramous, single segmented | uniramous, single segmented | uniramous, single segmented | uniramous, single segmented |
| **Leg 5: setation** | two setae | three setae | one dorsolateral seta of fifth pedigerous somite and three unequal setae on free segment | three equal setae | two setae |
